# Organic–Inorganic Hybrid Nanomaterials

**DOI:** 10.3390/nano9091197

**Published:** 2019-08-26

**Authors:** Valentine P. Ananikov

**Affiliations:** Zelinsky Institute of Organic Chemistry, Russian Academy of Sciences, 119991 Moscow, Russia; val@ioc.ac.ru

**Keywords:** nanomaterials, hybrid materials, nanoparticles, nanosalts, catalysis, organic synthesis, self-assembling, self-organization, bioconjugation, polymers, biomacromolecules, nanocomposite

## Abstract

The paramount progress in the field of organic–inorganic hybrid nanomaterials was stimulated by numerous applications in chemistry, physics, life sciences, medicine, and technology. Currently, in the field of hybrid materials, researchers may choose either to mimic complex natural materials or to compete with nature by constructing new artificial materials. The deep mechanistic understanding and structural insight achieved in recent years will guide a new wave in the design of hybrid materials at the atomic and molecular levels.

Hybrid nanomaterials contain two or more different components, typically inorganic components (metal ions, metal clusters or particles, salts, oxides, sulfides, non-metallic elements and their derivatives, etc.) and organic components (organic groups or molecules, ligands, biomolecules, pharmaceutical substances, polymers, etc.) that are brought together by specific interactions which result in the synergistic enhancement of their functional properties. A hierarchy of interactions may be involved in the construction of hybrid materials, from the building of molecules (covalent bonds, π-complexation, etc.) to nanoscale binding and self-assembly (a wide variety of intermolecular interactions, including electrostatic interactions, dispersion interactions, H-bonding, etc.) and microstructuring (cooperative interactions in multiple modes). The combination of different components and structural layouts with different types of interactions results in a virtually infinite variety of unique task-specific materials.

In this short account, we survey recent achievements in the field of hybrid organic–inorganic systems, which is the subject of this Special Issue. A detailed review of the literature in such a restricted format would be impossible; instead, a selection of bright representative examples and their applications are highlighted.

The design of hybrid nanomaterials, for example the development of new platforms for drug delivery and of stimuli-responsive smart materials and sensors, provides an outstanding driving force for the rapid progress in several research areas including nanomedicine, industrial technologies, materials science, and energy applications. The generation of fundamental knowledge in a particular field of science quite naturally empowers breakthrough projects in other fields. For instance, the quest for “cold fusion” in physics, which had admittedly failed to achieve its initial goal, was recently reported to advance the research in the field of highly hydrided metals [1]. A comprehensive revisiting of the experiment yielded improved PdH_x_ materials formed upon the absorption of hydrogen by palladium. These findings are highly valuable for the fields of catalysis and electrochemistry, given the unique performance of PdH_x_ palladium hydrides in reduction processes.

Hybrid organic–inorganic materials are powerful mimics of natural structures. These challenging natural composites achieve their properties through a complex, million-year-evolution-optimized hierarchical assembly, which is extremely difficult to reproduce. A recent study has demonstrated that a particular combination of two well-known compounds (aluminum oxide as the inorganic component and polymethyl methacrylate as the organic component) in a hybrid structure emulates natural toughening mechanisms to achieve high strength and fracture toughness of ~200 megapascals (MPa) and ~30 MPa·m^1/2^ [2].

Combining the concepts of nanotechnology with powerful synthetic methodologies based on supramolecular chemistry and the principles of self-assembly/organization opens a possibility to access higher order functional materials for the construction of nanoscale devices and machines [3,4]. The concept of nanoarchitectonics has emerged as a result of interdisciplinary initiatives at the intersection of nanotechnology, organic chemistry, life sciences, and biotechnology [3,5]. The next possible level of complexity has been suggested by the discovery of enzyme stabilization [6], thus bringing forward the idea of incorporating entire enzyme molecules into nano-/micro-sized hybrid materials. Responsive bioconjugate materials, a crossbreed of responsive polymers and biomacromolecules, facilitate the development of new synthetic tools with several advanced proofs of concepts, approaching the level of commercial implementation [7].

In hybrid materials, the organic component ranges from simple organic molecules to advanced molecular architectures [8] and organic polymers [9,10]. The inorganic part of hybrid materials, preferably tunable at the atomic level, may involve various inorganic moieties, from monometallic species to clusters and nanosized inorganic particles up to extended phases [9,11,12]. The functionalization of organic polymers with inorganic components represents an easy and convenient way for the development of innovative properties [9,10]. 

Organic units used as building blocks for hybrid polymeric materials are amazingly modifiable. As far as inorganic units are concerned, hybrid materials open other opportunities and allow the construction of coordination polymers with metals directly incorporated in the polymer chains and frameworks [13,14,15]. The flexible nature of the metal–ligand coordination bonds is central for the capability of adaptive recognition of chemical environments, which ensures dynamic selectivity towards particular reactants in complex mixtures [13].

Coordination bonds between metal centers and heteroatomic (S, Se, O, N, etc.) organic ligands generate specific polarized “nanosalt” structures [16] strikingly different from the regular non-hybrid structures that rely on metal–metal binding and lead to ordinary metallic nanoparticles. A high degree of polarization and the involvement of ionic species may be also achieved by using salt solutions [17] including the utmost extension to concentrated “solvent-in-salt” systems [18].

In addition, a large number of organic–inorganic hybrid materials have been developed in the course of various innovative projects dealing with photoactive devices [19,20], photocatalysis [21], high-performance electrochemical capacitors [22], and solar cells [23,24]. Hybrid materials occupy a unique niche between regular nanomaterials and nanocomposites, which enriches their structural motifs [25]. Controllable porosity and exposure to surface functional groups promote the use of hybrid materials for such challenging applications as gas sensing and capture [26,27,28].

A number of functional nanostructured materials with promising electrical, optical, thermal, and mechanical properties have been developed based on graphene, fullerene, and carbon nanotubes. Carbon materials and their heteroatom-doped derivatives provide an excellent starting point for the production of advanced materials with tunable properties [29,30] to be used for energy storage [31,32], electrocatalysis [33], and in optoelectronic devices [34].

The field of catalysis is continuously bubbling with the cutting-edge development of organic–inorganic nanoscale hybrid systems [35,36,37,38,39,40]. The quest for higher activity, better selectivity, and improved stability of catalysts is increasingly extended to the emerging hybrid organic–inorganic structures. The physical diversity of catalytically active centers, which goes to the core of the concept of catalytic “cocktails” [41], is commensurate with the nature of hybrid materials, which have the capacity to simultaneously encompass monometallic species, metal clusters, and metal nanoparticles. The challenging issues of biological activity and toxicity of metal-containing catalysts are central for the development of sustainable eco-friendly technologies [42,43]. 

This Special Issue contains a collection of articles on organic–inorganic hybrid materials [44]. Cellulose nanocrystals obtained from the ordinary filter paper and grafted with titanium dioxide nanoparticles give rise to a drug delivery nanocomposite prepared by complexation of the drug triclosan [45]. With the retained antibacterial activity of triclosan against *Escherichia coli* and *Staphylococcus aureus*, the developed hybrid drug delivery platform demonstrated long-term release profiles. The presence of titania was crucial for the antibacterial activity of the obtained nanocomposites [45].

In another study, composite iron oxide/gold particles were prepared by the on-site reduction of an inorganic Au salt by polyethyleneimine attached to iron oxide nanoparticles [46]. The resulting hybrid material exhibited superparamagnetic properties and plasmonic response in a dark-field microscope, suggesting the possibility of a dual-imaging probe. Interestingly, the attachment of gold nanoparticles may significantly reduce the cytotoxicity of the obtained material [46]. A comprehensive understanding of the biological activity of metal nanoparticles is important for a number of reasons, e.g., to avoid overestimations of their toxicity [42,43].

Hybrid materials based on nanocrystalline semiconductor matrixes, SnO_2_ or In_2_O_3_, and heterocyclic Ru (II) complex as a photosensitizer were studied as gas sensors operating under photoactivation with visible light at room temperature [47]. The sensitivity indicated by functional measurements was sufficient to detect 0.25–2 ppm of NO_2_. The role of the organic dye in the hybrid structure was to shift the photosensitivity range towards the low energies (longer wavelengths).

The translation of molecular-level interactions to the nanoscale level, followed by the specific assembling of microscale morphology, was reported for supramolecular organogels [48]. The study shows a novel example of aerogel prepared from a supramolecular gel, with a molecular-level understanding of the bottom-up assembling mechanism rarely achieved in structural studies.

A new approach for post-modification and mastering of carbon materials was developed using metal nanoparticles under microwave treatment [49]. A systematic study involving various metal-containing composite particles, including oxides, carbides, and neat metal systems, was carried out. The microwave treatment resulted in different visual appearances—single spark discharge, high-temperature red heat state or glow-of-plasma effect. The concomitant structural changes in the carbon surface depended on the type of metal-containing particles and treatment conditions.

Catalytic systems can be significantly improved by the advantageous application of hybrid structures. Intermetallic PdIn nanoparticles were supported on alumina, giving a Pd_1_In_1_/α-Al_2_O_3_ catalyst, which was evaluated for selective liquid-phase hydrogenation of diphenylacetylene [50]. Efficient kinetic control of the hydrogenation process and excellent reaction selectivity were observed. The temperature of catalyst preparation was varied over the range of 200–600 °C to reveal the contributions of different intermetallic structures.

Polyphenols from tea extracts were used as double-function reducing and capping agents for the eco-friendly preparation of gold nanoparticles [51]. The synthesized gold nanoparticles efficiently catalyzed the reduction of various aromatic nitro compounds in aqueous solutions. A variety of experimental conditions and the possibility to re-cycle and re-use of the nanoparticle catalyst were examined.

To summarize, the discovery and development of hybrid organic–inorganic systems fill the gap between the synthesis of molecules/nanoparticles and their function. Nanoscale devices and machines, stimuli-responsive smart materials, new generations of high-performance catalysts, drug delivery systems, and biocompatible materials, energy research, photovoltaics, and light conversion/generation systems are impressive and represent examples of recent achievements. Research in the area of hybrid systems provides an outstanding driving force for the development of new technologies and will facilitate their commercialization in the near future.

In the field of hybrid materials, researchers may choose either to mimic complex natural materials or to compete with nature by constructing new artificial materials. Both opportunities shape the upmost border of modern cutting-edge science. Currently, we are only at the beginning of the era of hybrid organic–inorganic systems. The deep mechanistic understanding and structural insights achieved in recent years will guide a new wave in the design of hybrid materials at the atomic and molecular levels.
